# Activity Blockade and GABA_A_ Receptor Blockade Produce Synaptic Scaling through Chloride Accumulation in Embryonic Spinal Motoneurons and Interneurons

**DOI:** 10.1371/journal.pone.0094559

**Published:** 2014-04-14

**Authors:** Casie Lindsly, Carlos Gonzalez-Islas, Peter Wenner

**Affiliations:** Physiology Department, Emory University, School of Medicine, Atlanta, Georgia, United States of America; Institut National de la Santé et de la Recherche Médicale (INSERM U901), France

## Abstract

Synaptic scaling represents a process whereby the distribution of a cell's synaptic strengths are altered by a multiplicative scaling factor. Scaling is thought to be a compensatory response that homeostatically controls spiking activity levels in the cell or network. Previously, we observed GABAergic synaptic scaling in embryonic spinal motoneurons following *in vivo* blockade of either spiking activity or GABA_A_ receptors (GABA_A_Rs). We had determined that activity blockade triggered upward GABAergic scaling through chloride accumulation, thus increasing the driving force for these currents. To determine whether chloride accumulation also underlies GABAergic scaling following GABA_A_R blockade we have developed a new technique. We expressed a genetically encoded chloride-indicator, Clomeleon, in the embryonic chick spinal cord, which provides a non-invasive fast measure of intracellular chloride. Using this technique we now show that chloride accumulation underlies GABAergic scaling following blockade of either spiking activity or the GABA_A_R. The finding that GABA_A_R blockade and activity blockade trigger scaling via a common mechanism supports our hypothesis that activity blockade reduces GABA_A_R activation, which triggers synaptic scaling. In addition, Clomeleon imaging demonstrated the time course and widespread nature of GABAergic scaling through chloride accumulation, as it was also observed in spinal interneurons. This suggests that homeostatic scaling via chloride accumulation is a common feature in many neuronal classes within the embryonic spinal cord and opens the possibility that this process may occur throughout the nervous system at early stages of development.

## Introduction

Homeostatic synaptic plasticity is the process by which neurons maintain cellular or network activity levels through compensatory adjustments of synaptic strength [1], [2], [3], [4]. For example, cultured neurons that experience days of activity blockade demonstrate an increase in the amplitude of excitatory miniature postsynaptic currents (mPSCs) and a decrease in the amplitude of inhibitory GABAergic mPSCs. These changes in the distribution of mPSC amplitudes appeared to be scaled, in that they were related to control values through a multiplicative scaling factor [5].

Previous work has demonstrated that blocking spike activity in the chick embryo *in ovo* results in GABAergic synaptic scaling in spinal motoneurons [6]. 48-hour infusion of a voltage-gated sodium channel blocker (lidocaine) induced an increase in GABA_A_ mPSC amplitude, which was compensatory due to the depolarizing nature of GABA at this stage of development. Further investigation demonstrated that lidocaine-induced GABAergic scaling was produced by a depolarizing shift in the GABA reversal potential mediated by chloride accumulation [7]. In addition to activity-block, *in ovo* block of GABA_A_ receptors (GABA_A_Rs) also produced an upward scaling of GABAergic mPSC amplitude in spinal motoneurons [8]. This finding suggested the possibility that GABAergic transmission is a critical step in the process of homeostatic plasticity, such that activity blockade reduces GABA release and therefore GABA_A_R activation, which then triggers synaptic scaling [9]. If this hypothesis is true, we would predict that activity-block and GABA_A_R-block should induce scaling via a common mechanism - a shift in the chloride reversal potential.

Homeostatic synaptic scaling has been widely studied in excitatory output cells (e.g. pyramidal cells), however inhibitory and excitatory interneurons have received far less attention. The studies that have looked for scaling in what is a highly diverse population of interneurons have had varied results depending on the particular interneuron studied [10]. However, in the embryonic chick spinal cord all interneurons whether they are GABAergic or glutamatergic are functionally excitatory, as are motoneurons. Thus, we hypothesized that following reductions in spiking activity or GABA_A_ transmission, all embryonic spinal neurons (interneurons and motoneurons) would compensate by accumulating chloride.

In order to ask these questions we have developed an imaging technique utilizing the ratiometric chloride indicator, Clomeleon [11], to assess intracellular chloride levels (Cl^−^_in_) in spinal neurons in the chick embryo. Since the Clomeleon protein is genetically encoded and is capable of labeling many neurons within a single spinal cord, this technique allows us to measure intracellular chloride levels faster and less invasively than is possible using intracellular recording. We find that intracellular chloride levels were higher in both motoneurons and interneurons after 24–48 hours of GABA_A_R block. The results support the hypothesis that the GABA_A_R is part of the machinery that triggers scaling and that this form of homeostatic plasticity is expressed throughout the embryonic spinal network.

## Methods

### Transfections (in ovo electroporations)

**Figure 1:**
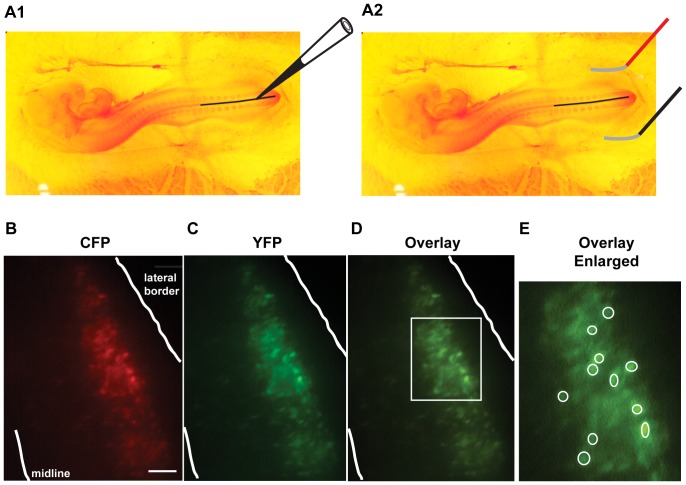
Genetic expression of Clomeleon in embryonic chick spinal motoneurons. **A)** Representation of Clomeleon transfections in chick neural tube at E3 (stage 14–15 [12]). **A1)** Plasmids coding for the Clomeleon protein were injected into the central canal of the neural tube. **A2)** Two electrodes spaced 4 mm apart were lowered onto the chorioallantoic membrane on either side of the embryo and electroporation pulses were delivered. **B&C)** Image of E10 spinal cord motoneurons expressing Clomeleon, visualized through the ventral white matter using a CFP illumination filter and CFP emission filter **(B)** or YFP emission filter **(C)** demonstrate clear co-labeling. Scale represents 100 µm. **D)** Overlay of the CFP and YFP images shows that the YFP (green) fluorescence is stronger than CFP (red) fluorescence, resulting in ratios (YFP/CFP) that are >1. **E)** Enlarged outlined rectangle from D. Circles indicate motoneuron soma regions of interest in which both CFP and YFP mean intensities were measured.

Typically on embryonic day 3 (E2–3, stages 14–15 [12]), a small window was cut into the shell of white leghorn chicken eggs (Hy-line North America LLC) and plasmids coding for the Clomeleon protein under control of a CMV promoter (kind gift of George Augustine) was injected into the central canal of the neural tube (depicted in Fig 1 A1). Two electrodes spaced 4 mm apart were lowered onto the chorioallantoic membrane on either side of the embryo and 5 pulses (25 V, 50 msec, interval of 1 sec) were delivered using an ECM 830 electroporator [13] (Fig 1 A2).

### Dissections

On E10, embryos were removed from the egg and the lumbosacral region of the spinal cords with attached spinal nerves were isolated as previously described [14]. Dissections were performed in 300 mls of filtered recirculating 15°C Tyrode's solution. The solution contained the following (in mM): 139 NaCl, 12 D-glucose, 17 NaHCO_3_, 3 KCl, 1 MgCl_2_, and 3CaCl_2_. After isolation of the spinal cord, the solution was replaced with fresh Tyrode's solution (300 mls) and warmed to 17°C. Spinal cords were left overnight at 17°C so that recovery of spontaneous network activity (SNA), which takes several hours, would be complete before the experiment began. Before the cords were moved to the recording chamber, the pia mater was removed from the ventral side of the cord for improved visualization and the bath solution was brought up to room temperature. None of the Tyrode's solutions used contained drugs that were injected *in ovo*.

### Clomeleon Imaging

**Figure 2:**
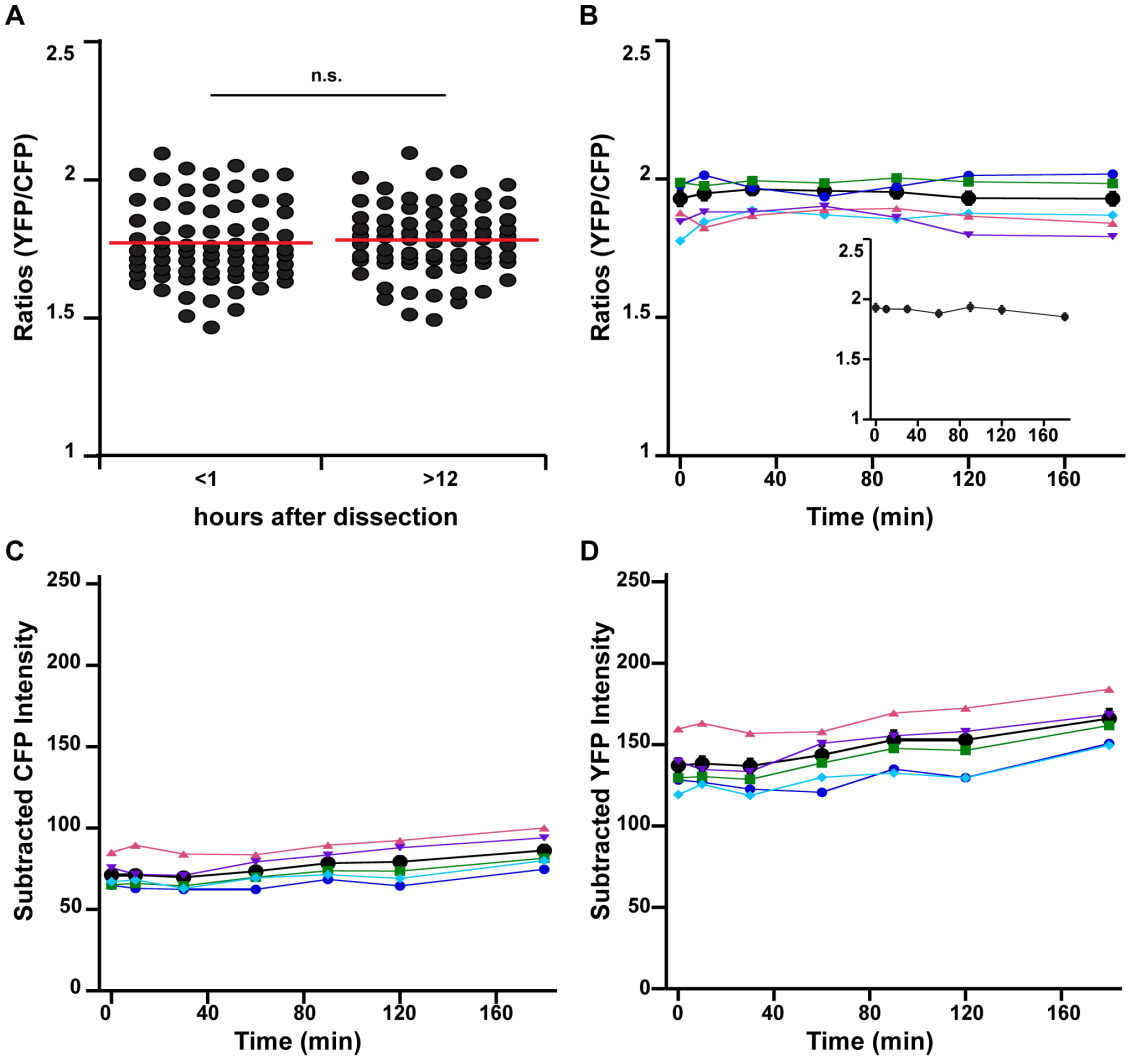
Clomeleon labeling in the chick spinal cord provides stable FRET ratios over time. **A)** FRET ratios for motoneurons <1 hour after dissection, and >12 hours after dissection at room temperature. For 7 cords (70 cells) there was no statistical difference between the means at each time period (p = 0.1) indicating stability of the signal. Red lines indicate average values. **B)** Ratios measured over 180 minutes in 27°C Tyrode's solution indicating the stability of the average ratios of 10 cells from 1 cord (black) and for ratios of 5 individual cells (matching colors for B, C, and D). Inset: Average ratios over 180 minutes for 40 cells from 4 cords. **C)** Subtracted CFP intensity from same 5 cells (avg in black) as in B. **D)** Subtracted YFP intensity from same 5 cells (avg in black) in B. The stability of the ratios and subtracted CFP and YFP values suggest our imaging methods produce minimal photobleaching.

Cords were placed ventral side down in the recording chamber of an Olympus IX70 inverted microscope and were continuously perfused with fresh Tyrode's solution (∼50 mls). The solution was heated to 27°C for all experiments except those in Figure 2A, which were conducted at room temperature. Transfected neurons were imaged through the ventral white matter using a 10× objective. Clomeleon is a fusion protein containing 2 fluorophores, the Cl^−^ -insensitive CFP (cyan fluorescent protein), and the Cl^−^ -sensitive YFP (yellow fluorescent protein). Illumination results in excitation of CFP (430–450 nm), producing emission at 485 nm, which excites the YFP fluorophore through fluorescence resonance energy transfer (FRET). Thus, emitted light was passed through a dichroic mirror with a 460 nm cutoff and then filtered through emission filters for CFP (485±15 nm) or YFP (530±15 nm). In order to limit differential photobleaching of the Clomeleon fluorophores, we used neutral density filters so that only 2–6% of light reached the specimen. In addition, we limited exposure time using a uniblitz shutter (Vincent Associates, Rochester). The emission for each fluorophore was then captured onto an intensified CCD camera (Stanford Photonics) and images were recorded using simple PCI software (Hamamatsu) as a 20 frame average. Images were then processed in Simple PCI by measuring the mean intensity of a region of interest (ROI) drawn around a cell body (Fig 1E) and subtracting the mean intensity for a background ROI located in a non-labeled part of the cord. This process was conducted for both CFP and YFP images and the subtracted values were expressed as a ratio (YFP/CFP) for each cell. Since Clomeleon is sensitive to internal pH, we monitored and maintained the pH of the bath at 7.2–7.3 during recordings.

### Calibrations

**Figure 3:**
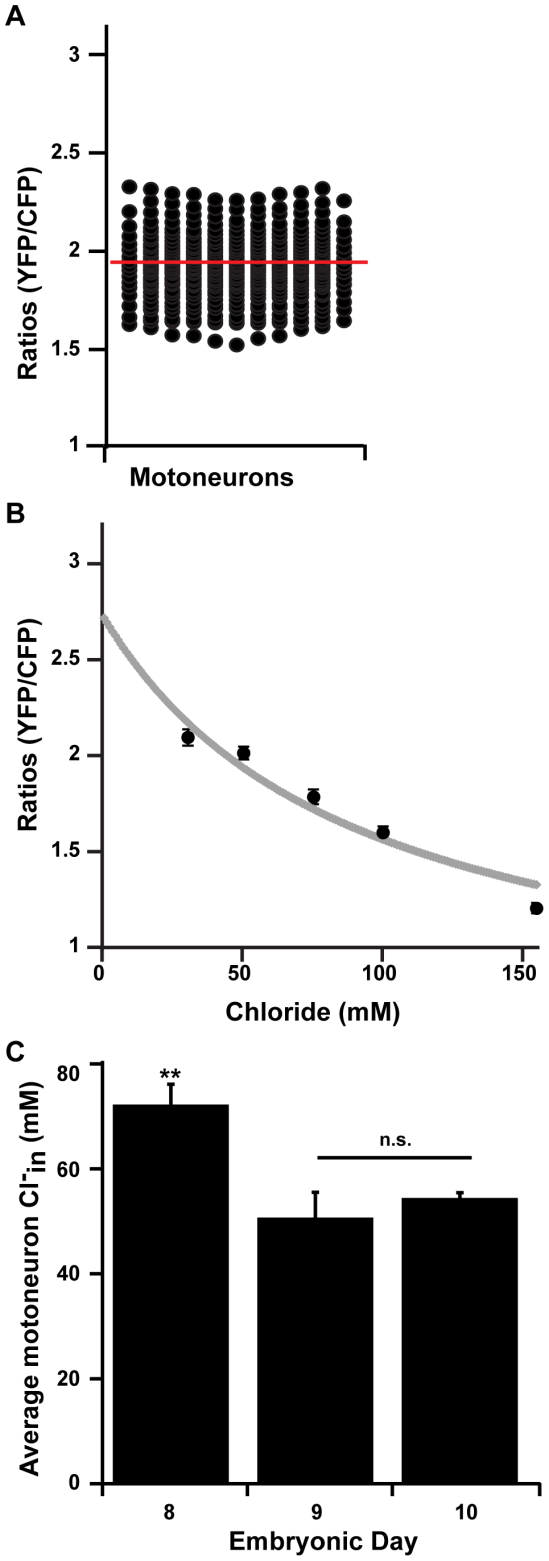
Clomeleon measurements confirm high levels of Cl-in in spinal motoneurons. **A**) Scatter plot demonstrating the range of FRET ratios measured from motoneurons from 33 chick spinal cords (E10). The average from 330 motoneurons is 1.92±0.01, and is indicated by the red bar. **B)** Graph indicating the average ratios from the calibration experiments (black dots, n = 5 cords, error bars represent S.E.M.) as well as the relationship between the calibration ratios and Cl^−^_in_ for somas (grey line, see methods for details). **C)** When ratios measured from motoneuron somas are converted to Cl^−^_in_, we find that the average at E8 is significantly higher than at E9 or E10 (p<0.001). However, values at E9 and E10 are not significantly different (n.s., p = 0.97).

In order to calibrate the fluorescence of Clomeleon on our imaging system, we measured the FRET ratios in the following solutions: 150 mM Cl^−^, 75 mM Cl^−^, 50 mM Cl^−^, 30 mM Cl^−^ (chloride and gluconate concentrations summed to 150 mM). In some cases, an individual cell or cells had ratios of ∼1 and did not respond to changes in solutions. These cells were presumed to be unreliable; therefore, they were not used in the data sets. All calibration solutions contained 10 µM nigericin (K^+^/H^+^ ionophore) and 100 µM tributyltin chloride (Cl^−^/OH^−^ antiporter) to remove transmembrane H^+^/OH^−^ and Cl^−^ gradients [15]. The dissociation constant (K_D_) and R_max_ (value for Clomeleon completely unbound by Cl^−^) were determined from a non-linear regression of the average ratios to [Cl^−^] using the following equation: R = [(K_D_*R_max_)+([Cl^−^]*R_min_)]/([Cl^−^]+R_min_) [16]. R_max_ could not be evaluated from a 0 mM Cl^−^ calibration solution as ratio values dropped, and did not recover in other calibration solutions. The R_min_ (value for Clomeleon completely bound by Cl^−^) was determined from ratios measured in a KF solution as F^−^ is known to saturate the YFP moiety. Upon testing 5 spinal cords we found, R_min_ = 0.53±0.01, K_D_ = 91.6±27.0, and R_max_ = 2.7±0.28 (Fig 3B). These values allowed for conversion of ratios to Cl^−^_in_ using the following formula: Cl^−^_in_ = K_D_ (R_max_−R)/(R−R_min_)[15], [16], [17].

### Texas Red fill and imaging

For labeling motoneurons with Texas red, the ischiadic plexus was drawn into a suction electrode just after dissection. Most of the Tyrode's solution was removed from the electrode and a small amount of Texas red (dissolved in dH_2_O) was injected into remaining solution and left to fill overnight. After the bath solution reached room temperature, the cord was moved to the vibratome (Leica VT1000S) where transverse slices were made from the lumbosacral cord. Slices were then moved to an Axio Vert Zeiss microscope. Labeled motoneuron somas and dendrites were visualized using a Rhodamine filter. Images were then taken with QCapture and processed with ImageJ.

### *In ovo* Pharmacology

For chronic activity blockade, an aqueous solution of lidocaine hydrochloride (Sigma) (135 mM solution adjusted to pH 7) was continuously applied onto the chorioallantoic membrane of the embryo at a rate of 13.5 µL/h from E8 to E10, as previously described [6]. For blockade of GABA_A_R transmission, 50 µL of a 10 mM gabazine solution (Tocris, 10 µM assuming an egg volume of 50 ml) was injected onto the chorioallantoic membrane of the embryo [8] on E8, E9, or E9.5 for 48 h, 24 h, or 12 h block, respectively. Chronic drug application was discontinued during both the dissection and imaging experiments so that the drugs would not be active during imaging experiments.

### Electrophysiology

Whole-cell patch-clamp recordings were made from spinal motoneurons localized in lumbosacral segments 1–3 to assess mPSCs, as described previously (Gonzalez-Islas et al., 2010). Briefly, whole-cell recordings were obtained from motoneurons (electrodes, 5–10 MΩ). Recordings were terminated whenever significant increases in input resistance (≥20%) occurred. Extracellular solution for mPSC recordings was Tyrode's solution described above plus TTX (1 µM), AMPA receptor antagonist CNQX (10 µM) and NMDA receptor antagonist APV (50 µM). The intracellular patch solution for these experiments contained the following (in mM): 5 NaCl, 100 K-gluconate, 30 KCl, 5 CsCl, 10 TEA-Cl, 10 HEPES, 1 MgCl2, 0.1 CaCl2, 1 Na2ATP, 0.1 MgGTP. Pipette solution osmolarity was between 280 and 300 mOsm, and pH was adjusted to 7.3 with KOH. Junction potentials were corrected online. Currents were low-pass filtered online (5 kHz), digitized at 10 kHz and analyzed with Mini Analysis (Synaptosoft).

For evoked ventral root potential recordings, two adjacent ventral roots were taken up into tight-fitting suction electrodes connected to extracellular amplifiers (A-M Systems). Stimulation (15 µA, 0.5 msec) was delivered to one electrode using a Master 8 system and an ISO-Flex stimulus isolator (A.M.P.I), while recording the potential from the adjacent root using an AC/DC differential amplifier (A-M Systems Inc., digidata1322A and pClamp9 software, Molecular Devices). Solutions during ventral root recordings consisted of a Tyrode's solution described above with, AMPA receptor antagonist CNQX (10 µM) and NMDA receptor antagonist APV (50 µM).

### Statistics

FRET ratios (R) were taken from ∼10 motoneurons/cord, in order to avoid bias toward cords with the highest labeling density. The labeling of interneurons was more sparse, therefore all neurons were analyzed and only average cell statistics are presented. Data from cell and/or cord averages are expressed as the mean ±S.E.M. Unpaired student's T-tests or ANOVAS with Bonferroni post hoc test were conducted in IBM SPSS Statistics Program and used for statistical analysis where appropriate. Significance was established based on a p value <0.05. Linear and non-linear regression analyses were conducted in IBM SPSS Statistics Program.

## Results

### Clomeleon provides reliable approximation of baseline Cl^−^_in_ for spinal motoneurons

Previous results using gramicidin perforated patch, or whole cell recordings have shown that E10 chick spinal motoneurons maintain high levels of intracellular chloride (∼50 mM when external Cl^−^ is 150 mM; [7], [18], [19]. In order to obtain measures of Cl^−^_in_ from a large number of chick spinal neurons in a less invasive manner than by intracellular recording, we used the fluorescent ratiometric chloride indicator, Clomeleon. The expression plasmid carrying the coding region of Clomeleon was injected into the central canal of the chick neural tube at E2–3. The plasmid was then electroporated into spinal neurons on one side of the cord and eggs were returned to the incubator to develop for another week. After isolation of the spinal cord at E10, we found robust Clomeleon expression that was visible through the ventral white matter using CFP illumination and acquiring the emitted light for either CFP or YFP (Fig 1B–E). Following electroporations at E3 most of the labeled cells occupied a lateral position in the hemicord. It has been known for many years that the vast majority of the cells occupying the lateral part of the ventral spinal cord in the embryonic chick (lateral motor column) are motoneurons [20], [21]. Clomeleon-expressing cells that had large cellular profiles and occupied a lateral position were chosen for analysis and were assumed to be motoneurons.

Background-subtracted fluorescence intensity was measured in both the YFP and CFP channels for approximately 10 somas per cord. Examples of somatic regions of interest (ROIs, white circles) can be seen in Figure 1E. Measurements of background intensity were taken from an ROI on the non-labeled side of the cord. The mean intensity for the background region was subtracted from the mean intensity for the somatic regions for both YFP and CFP. FRET ratios (YFP subtracted intensity/CFP subtracted intensity) for each cell were then determined in multiple cords.

We carried out experiments to determine the stability of the ratios over time. Ratios measured just after the dissection (<1 h = 1.70±0.02, n = 70 cells from 7 cords) were not different from ratios taken from the same cords the next morning (>12 h = 1.74±0.02, n = 70 cells from 7 cords, p = 0.1, Fig 2A). The <1 h and >12 h ratios were taken at room temperature to avoid warming the preparation several hours before carrying out the following days experiment; however, all subsequent ratios were taken at 27°C. Temperature affected Clomeleon ratios/Cl^−^_in_ (compare Fig 2A and Fig 3A). Additionally, since the YFP and CFP fluorophores of the Clomeleon protein are subject to differential photobleaching, we measured the YFP/CFP ratios over several hours to ensure the consistency of the ratios throughout longer experiments. We found the ratios and subtracted intensities for YFP and CFP were stable over 3 hours (Fig 2B–D), and in one cord we measured ratios for an extended period and found that ratios were stable for ≈7 hours (data not shown). Figure 2B–D shows data from 5 individual cells taken from a single representative cord (only 5 shown for clarity), and the average of the 10 cells in that cord (black, Figure 2B–D); the average from 4 cords (10 cells each cord, Figure 2B inset) further shows stability of the ratios over time. These data indicate that our imaging parameters (described in methods) do not produce differential bleaching over this period.

Calibration experiments were carried out in order to convert ratios to approximate chloride concentrations (see methods). Upon measuring FRET ratios and converting to Cl^−^, we found that the Cl^−^_in_ for spinal motoneurons at E10 was 54.7±1.0 mM (n = 330 cells from 33 cords, Fig 3). This concentration is similar to values that were obtained using perforated patch and whole cell techniques [7], [18], [19], strengthening our confidence in the optical technique. It has been shown in cultured hippocampal neurons that Clomeleon is capable of capturing the shift of baseline Cl^−^_in_ from ∼140 mM at E18 to ∼20 mM at P14 [11]. We tested if Cl^−^_in_ for spinal motoneurons was changing between E8 and E10 (timing of drug treatments below). We found that there was a developmental shift of Cl^−^_in_ for spinal motoneurons (Fig. 3C; E8 - 72.5.1±4.0 mM, n = 34 cells from 4 cords; E9 - 50.9±4.9 mM, n = 29 cells from 3 cords; E10: 54.7±1.0 mM, n = 330 from 33 cords, p<0.001).

### Chronic activity-block and GABA_A_R-block produced similar increases in baseline motoneuron Cl^−^_in_

**Figure 4:**
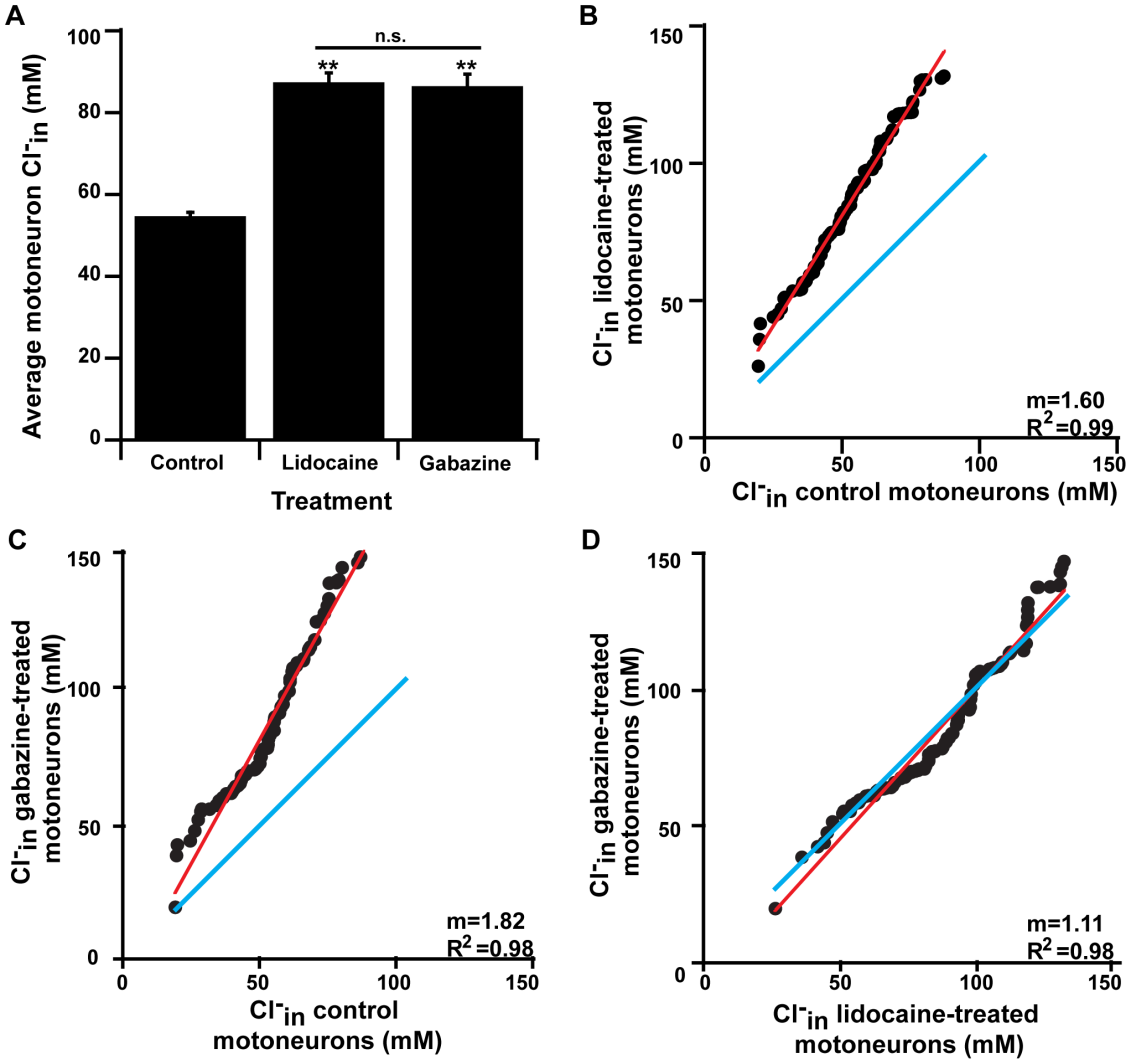
Spinal motoneurons exhibit a similar increase in chloride accumulation after chronic activity block or chronic GABA_A_R block. **A**) Bar graph indicating Cl^−^_in_ in spinal motoneurons is significantly greater after either chronic lidocaine or gabazine treatment compared to control motoneurons (** indicates treatment vs. control Bonferroni's p<0.001). Average Cl^−^_in_ after chronic gabazine is not significantly different (n.s.) from the average Cl^−^_in_ after chronic lidocaine (Bonferroni's p = 1). **B**) Graph of ranked distribution of Cl^−^_in_ for motoneurons from control cords vs. cords treated with lidocaine. Red line indicates linear relationship, blue line represents line of unity. **C**) Graph of ranked distribution of Cl^−^_in_ for motoneurons from control cords vs. cords treated with gabazine. Red line indicates linear relationship, blue line represents line of unity. **D**) Graph of ranked distribution of Cl^−^_in_ for motoneurons from lidocaine treated cords vs. Cl^−^_in_ for motoneurons from gabazine treated cords. Red line indicates linear relationship, blue line represents line of unity. The same number of cells are plotted from gabazine-treated, lidocaine-treated, and control cords for B–D (control cells are the same for B & C).

Previous studies blocking spontaneous network activity by infusing lidocaine into the egg for 2 days triggered synaptic scaling of GABAergic mPSCs that were mediated by chloride accumulation [6], [7]. To confirm these results, we treated embryos with lidocaine as described previously. Following *in vivo* treatment we isolated the spinal cord, no longer in the presence of lidocaine. As reported previously [6], the inter-episode interval between episodes of SNA in the isolated cord was significantly reduced in lidocaine treated embryos compared to controls (control – 616±25 sec, n = 12; lidocaine – 336±38 sec, n = 6; p<0.001). In spinal motoneurons, we found that 48-hour treatment of lidocaine *in ovo* from E8–10, resulted in significantly elevated Cl^−^_in_ compared to controls (Fig 4A; 87.4±2.4 mM, n = 106 cells from 11 cords; p<0.001). Together, these results support the hypothesis that chronic activity-block can produce scaling that is dependent on a shift in E_Cl_. To establish if the increases in Cl^−^_in_ were observed across the entire population of motoneurons, we expressed the ranked distribution of Cl^−^_in_ levels from lidocaine-treated cords as a function of the ranked order distribution of Cl^−^_in_ levels from control cords (Fig 4B). We found that these two distributions were linearly related with a slope greater than one, which suggested that 48-hour lidocaine treatment produced a multiplicative increase in Cl^−^ values across the population of motoneurons.

The sensing mechanism that triggers synaptic scaling has been widely debated in the field. In a previous study, we found that *in ovo* GABA_A_R blockade with gabazine or bicuculline (from E8–10) also resulted in an upward scaling of GABAergic mPSCs, but to a greater extent than lidocaine-treatment [8]. This finding was consistent with the idea that blocking activity with lidocaine triggered scaling because it reduced action potentials, which reduced spike-dependent GABA release and consequent GABA_A_R activation. It is unknown whether GABAR block triggers GABAergic scaling by increases in Cl^−^_in_. If both lidocaine and gabazine trigger scaling through a reduction in GABA_A_R activation, then we would expect that both drugs trigger scaling through increased chloride accumulation. To test this hypothesis we measured Cl^−^_in_ for motoneurons after *in ovo* treatment of 10 µM gabazine from E8–10. Following chronic *in ovo* gabazine treatment spinal cords were isolated from the embryo and gabazine was washed out via serial dilutions (see methods). The frequency of episodes of SNA in the isolated cord was not significantly different than controls (inter-episode intervals; control – 616±25 sec, n = 12; gabazine – 637±31 sec, n = 6). Clomeleon ratios were then obtained from spinal motoneurons and exhibited significantly higher Cl^−^_in_ compared to controls. Also, there was no difference in average Cl^−^_in_ between gabazine-treated and lidocaine-treated motoneurons (Fig 4A). Similar to lidocaine treatment, Cl^−^_in_ after gabazine treatment (E8–E10) was significantly higher than at E8 (E8 control - 72.5±4.0 mM, n = 34 cells from 4 cords; E10 gabazine-treated – 86.5±3.0 mM, n = 94 cells from 10 cords; p = 0.006). Based on the rank ordered plots of Cl^−^_in_ for gabazine-treated and control motoneurons, we again found a multiplicative increase of Cl^−^_in_ values across the population of motoneurons (Fig 4C). We also observed that the rank order distribution of Cl^−^_in_ for lidocaine-treated versus gabazine-treated motoneurons had a slope near 1 (blue line, Fig 4D). Together, the data supports the hypothesis that chronic GABA_A_R-block and activity block (lidocaine) produce scaling through a common depolarizing shift in E_Cl._ We carried out similar experiments with another GABA_A_ antagonist, bicuculline and obtained similar results (bicuculline 50 µM- 74.3±2.2 mM n = 50 cells from 5 cords ANOVA p = 0.001). Therefore, the results support the hypothesis that GABA_A_Rs are an important element in the sensing machinery that triggers GABAergic synaptic scaling in embryonic chick motoneurons.

**Figure 5:**
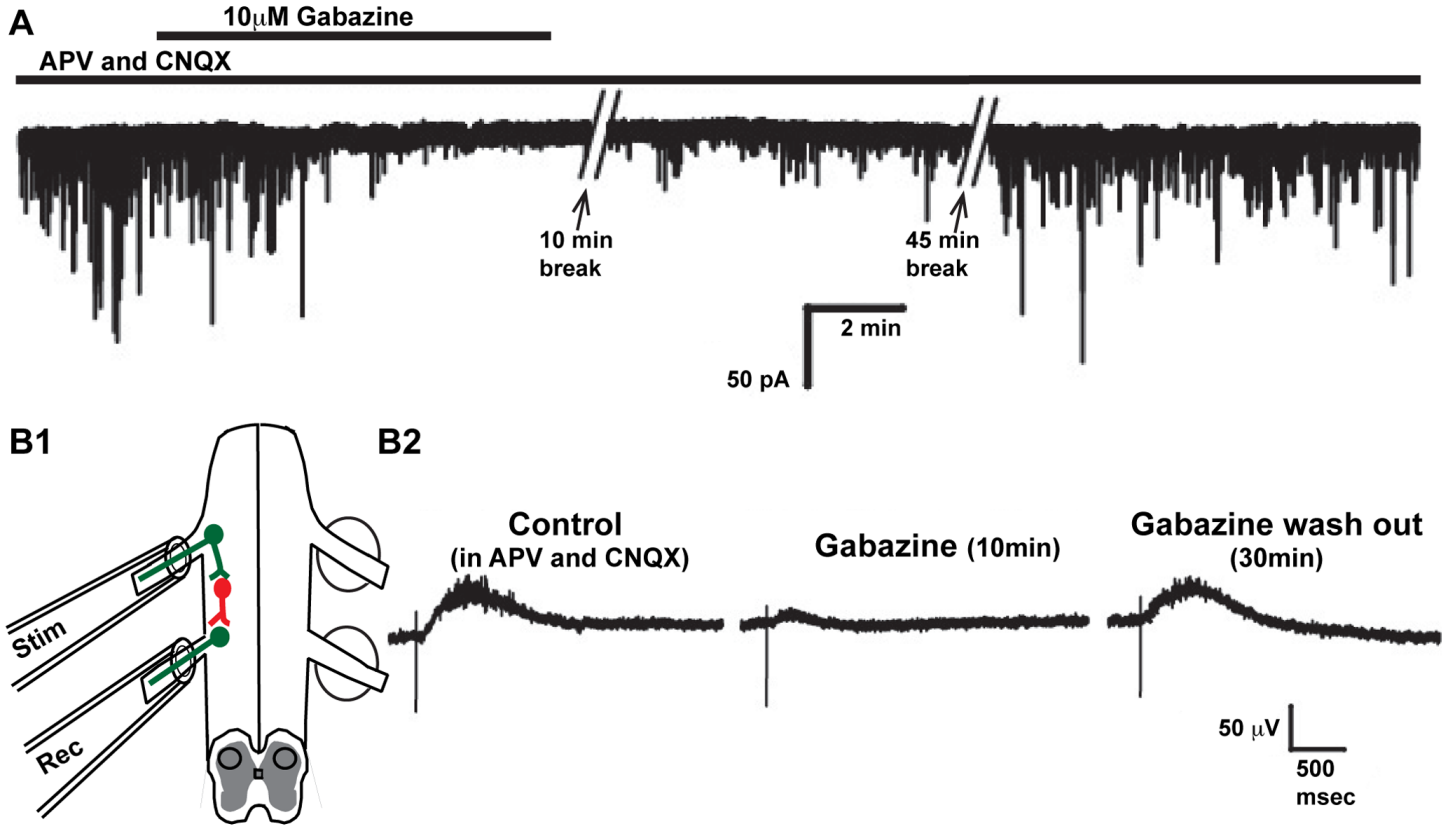
Gabazine is effectively washed out of the isolated spinal cord within minutes. **A**) Trace of GABAergic mPSCs recorded from a spinal motoneuron in the presence of CNQX (10 µM) and APV (50 µM). Gabazine (10 µM was added to the bath solution for 8 minutes (indicated by bar). During this time the mPSCs are dramatically reduced. Once gabazine is washed out of the solution the mPSCs return. Time stamps indicate the time between traces. **B1**) Schematic of an isolated spinal cord with two ventral roots taken into tight fitting suction electrodes. One electrode was used to stimulate the first ventral root while the second was used to record the potential in the adjacent ventral root. The circuit being stimulated is illustrated with green motoneurons and red R-interneurons [50]. **B2**) In the presence of CNQX and APV, stimulation of this circuit produces a GABAergic response. The amplitude of this response is dramatically reduced after a 10 min application of gabazine (10 µM After a 30 min wash out, the amplitude of the ventral root potential returns to baseline.

Another interpretation of the results could be that gabazine is not sufficiently washed out of the preparation, leading to altered Cl^−^_in_. We think this is unlikely as serial dilutions of Tyrode's solution (without gabazine) are carried out during both the dissection and the transfer to a recording chamber (see methods). Further, we find that gabazine readily washes out. GABAergic mPSCs from motoneurons were recorded using whole cell patch. These mPSCs were effectively eliminated with 10 µM gabazine and then began to recover within 10 minutes after washing out gabazine, and were completely recovered 60 minutes after the washout began (Figure 5A). In addition, we stimulated a ventral root while recording potentials from the adjacent ventral root (circuit described in Fig 5B1). This GABAergic response was eliminated in the presence of 10 µM gabazine and recovered after washing out gabazine for 30 minutes (Figure 5B2).

### Time-course of chloride accumulation in motoneurons

**Figure 6:**
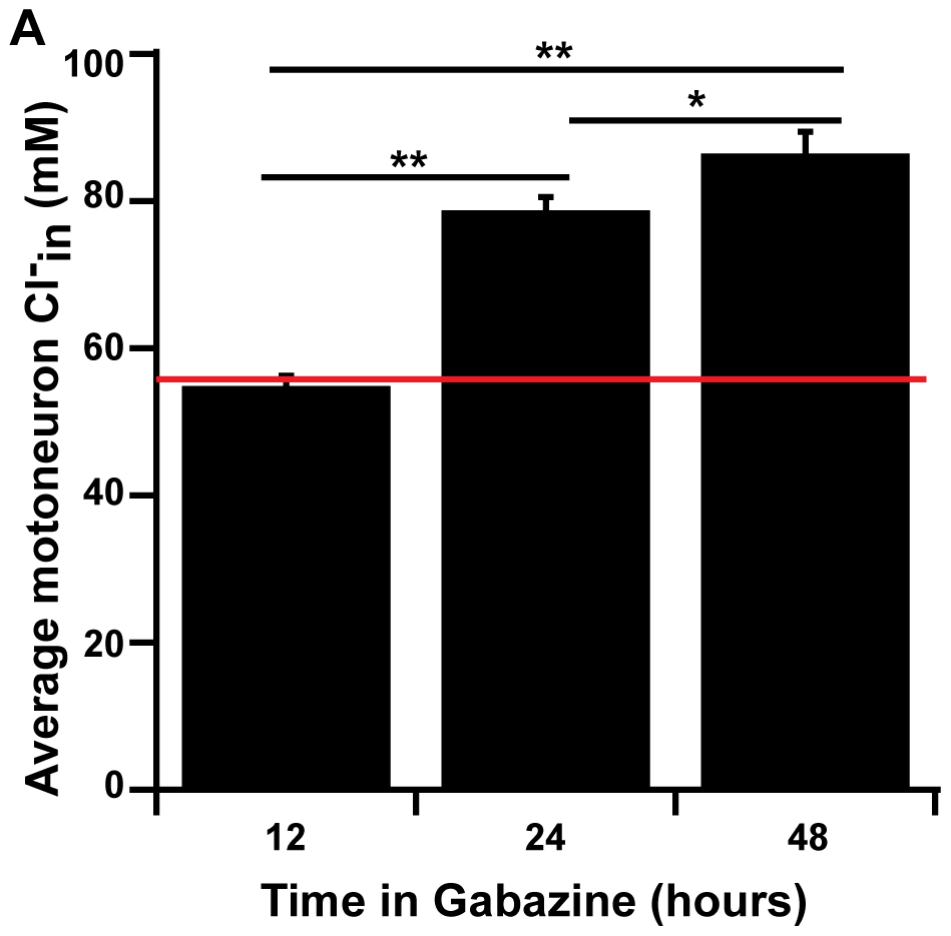
Timescale of the increase in Cl-in in spinal motoneurons after GABAAR blockade. A bar graph indicating the average Cl^−^_in_ for motoneurons from embryos treated with gabazine for 12, 24, or 48 hours. Red line indicates control value. Statistics: ANOVA p value<0.001; for Bonferroni's * indicates p = 0.03, ** indicates p<0.001. Not significantly different (n.s.). For numbers see Table 1.

One of the hallmarks of synaptic scaling is that it develops slowly, often requiring at least 24 hours of altered activity. Supporting this idea, previous work in the lab using whole cell recordings of mPSCs demonstrated that scaling occurred after 48 hours, but not after 12 hours of GABAergic blockade [8]. In order to better determine the time-course of GABAergic scaling, we assessed chloride levels via Clomeleon at E10, after 12 hours (from E9.5), 24 hours (from E9), or 48 hours (from E8) of gabazine treatment. In support of previous results, we found that after 12 hour treatment, Cl^−^_in_ was not different from non-treated controls. After 24 hour treatment there was an increase in Cl^−^_in_ that further increased after 48 hours of gabazine treatment (ANOVA p<0.001 for cell averages and cord averages; Figure 6 and Table 1).

**Table 1:** Cl^−^_in_ in spinal motoneurons and interneurons increases with longer durations of GABA_A_R block.

	Treatment	Cell Averages (mM)			Cord Averages (mM)		
		Cl^−^_in_	SE	n	Cl^−^_in_	SE	n
Motoneurons	E10 control	54.7	1.0	330	54.7	2.7	33
	12 h gabazine	54.8	1.5	82	54.7	2.5	9
	24 h gabazine	78.7	1.9	127	78.5	4.4	13
	48 h gabazine	86.5	3.0	94	86.4	7.0	10
	48 h bicuculline	76.3	2.2	50	76.3	1.7	5
Interneurons	E10 control	64.7	1.7	97			
	12 h gabazine	75.0	2.6	62			
	24 h gabazine	80.3	3.2	42			
	48 h gabazine	85.8	5.1	33			

Cl^−^_in_ for motoneurons and interneurons in control cords, and cords treated with gabazine for 12, 24, or 48 hours or treated with bicuculline for 48 hours. Cell averages for motoneuron and interneuron Cl^−^_in_ (mM) for all cells in a given condition, and n represents the number of cells. Cord averages for motoneurons represents the average of cord values in a given condition and n represents the number of cords. Cord averages for interneurons are not included as they were highly variable due to the variability of interneuron numbers per cord. Interneuron cord n's are: control  = 13, 12 h gabazine  = 7, 24 h gabazine = 6, and 48 h gabazine  = 5.

### Spinal interneurons and motoneurons express similar Cl^−^_in_ levels in control and treated embryos

**Figure 7:**
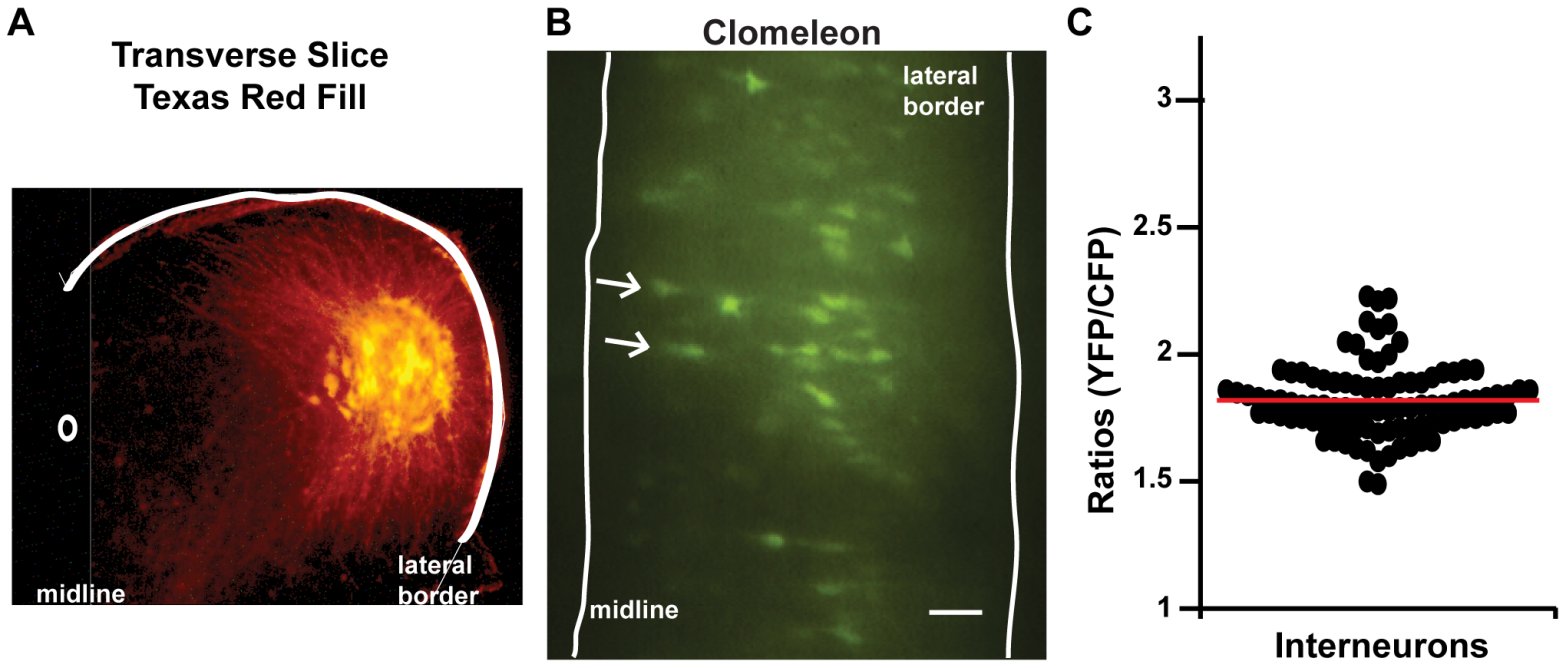
Spinal interneurons also exhibit high Cl-in. **A**) A transverse spinal slice showing Texas Red labeling of the lateral motor column (motoneurons) demonstrating their lateral location. **B**) A CFP-YFP overlay image of a ventral view of the E10 spinal cord expressing Clomeleon. The arrows indicate examples of spinal interneuron somas, which are identified as being clearly separate from the more lateral motoneuron column. Scale bar represents 100 µm. **C**) Scatter plot demonstrating the range of ratios measured from interneuron somas from 13 spinal cords. The average is indicated by the red line.

**Figure 8:**
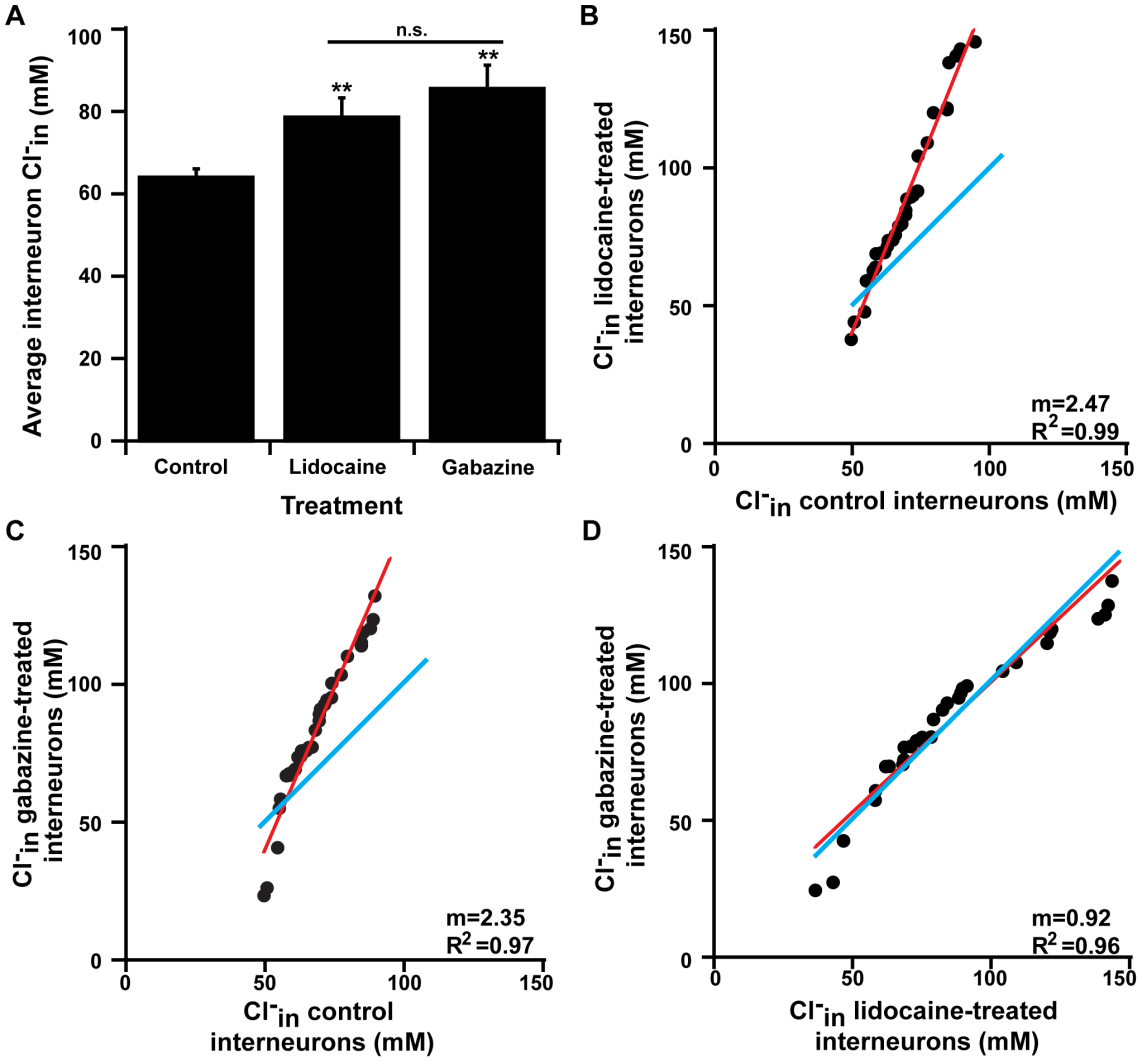
Interneurons also demonstrate increased Cl-in after block of both spiking activity and GABAergic transmission. **A**) A bar graph indicating that chloride accumulation in spinal interneurons was significantly greater after either chronic lidocaine or gabazine treatment compared to controls. Statistics: ANOVA p = 0.007; for treatment vs. control Bonferroni's * indicates p = 0.002, ** indicates p<0.001, error bars represent S.E.M., n.s. represents not significantly different. Average Cl^−^_in_ after chronic gabazine is not significantly different from average Cl^−^_in_ after chronic lidocaine (Bonferroni's p = 0.63). **B**) Graph of ranked distribution of Cl^−^_in_ for interneurons from control cords vs. cords treated with lidocaine. Red line indicates linear relationship, blue line represents unity. **C**) Graph of ranked distribution of Cl^−^_in_ for interneurons from control cords vs. cords treated with gabazine. Red line indicates linear relationship, blue line represents unity. **D**) Graph of ranked distribution of Cl^−^_in_ for interneurons from cords treated with lidocaine vs. cords treated with gabazine. Red line indicates linear relationship, blue line represents unity. The same number of cells are plotted from gabazine-treated, lidocaine-treated, and control cords for B–D (control cells are the same for B & C).

Motoneurons represent a fairly homogenous, highly accessible neuronal class, and as such have been the focus of many spinal cord studies. However, because Clomeleon also labels spinal neurons that are located medial to the lateral motor column, we can also assess Cl^−^_in_ for what is likely to be a diverse set of ventrally-located spinal interneurons (examples in Figure 7B, very few glia are present at this early stage in the ventral grey matter and glia exhibit different morphological profiles than neurons [22], [23], [24], [25]). We found that Cl^−^_in_ for control interneurons was higher than motoneurons (student's t-test p<0.001, Fig 7C, 8A, Table 1 for cells, and p = 0.04 for cords). This could be due to the developmentally younger state of spinal interneurons. Regardless, this finding demonstrates that GABAergic transmission at these early stages is depolarizing in both populations.

Previous studies of homeostatic scaling in interneurons have been highly varied. Because cholinergic, glutamatergic and GABAergic spinal neurons at embryonic stages are all functionally excitatory, we hypothesized that they may express homeostatic plasticity in a more homogeneous fashion. Therefore, we used Clomeleon labeling of interneurons to assess Cl^−^_in_ after chronic activity block *in ovo* (lidocaine injection). We found that following lidocaine treatment from E8–E10 there was a significant increase in Cl^−^_in_ compared to controls suggesting that scaling had been triggered (Fig 8A; control = 64.4±1.7 mM, n = 97 cells from 12 cords, Lidocaine treated = 78.9±4.3 mM, n = 48 cells from 8 cords; p = 0.002). Additionally, there was a multiplicative increase in Cl^−^_in_ across the interneuron population after lidocaine treatment, as we observed a linear correlation between the ranked order distribution of Cl^−^_in_ for interneurons from lidocaine-treated cords versus that of control (Fig 8B). This correlation suggests that there is a multiplicative increase in Cl^−^_in_ for what is likely to be a diverse population of interneurons.

**Figure 9:**
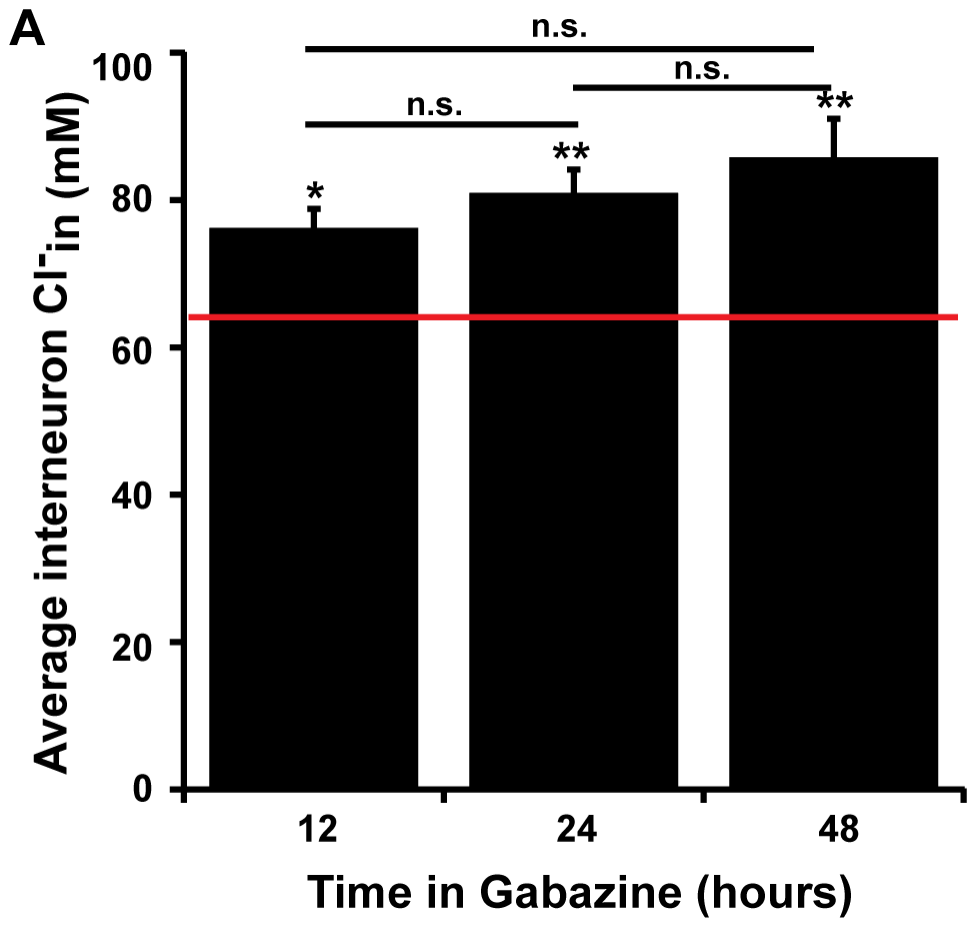
Interneuron chloride accumulation after different durations of GABAAR blockade. A bar graph indicating the average Cl^−^_in_ for interneurons after 12, 24, or 48 hours of gabazine-treatment. Red line indicates control value. Statistics: ANOVA p value<0.001; for Bonferroni's * indicates p = 0.006, ** indicates p<0.001, n.s. represents not significantly different, error bars represent S.E.M. For numbers see Table 1.

Furthermore, chronic block of GABA_A_Rs (gabazine) from E8–10 also produced a significant increase in Cl^−^_in_ compared to controls (ANOVA p<0.001, Fig 8A and Table 1). We also observed a multiplicative increase in Cl^−^_in_ across the population after gabazine treatment (Fig 8C). Increased Cl^−^_in_ for activity- and GABA_A_R-blocked interneurons were not significantly different (p = 0.06, Fig 8A); further, the ranked ordered distribution of Cl^−^_in_ for lidocaine-treated cords versus that of gabazine-treated cords was linearly related with a slope near 1 (Fig 8D). Again, these data indicate a similar mechanism between scaling after activity-block and GABA_A_R-block. We also wanted to assess chloride levels via Clomeleon at E10, after 12 hours (from E9.5), 24 hours (from E9), or 48 hours (from E8) of gabazine treatment. We found that Cl^−^_in_ was significantly increased from non-treated controls after all time points; however, there was no significant difference between Cl^−^_in_ after 12, 24, or 48 hour treatment (ANOVA p<0.001, Figure 9 and Table 1).

## Discussion

### Clomeleon imaging confirms chloride accumulation in embryonic motoneurons following activity blockade

Previously, we demonstrated that GABAergic mPSCs undergo a scaling up following *in ovo* activity blockade (lidocaine) through Cl^−^ accumulation using perforated patch and whole cell recordings [6], [7]. Further, following activity blockade there was no change in the conductance of GABAergic mPSCs suggesting there were not alterations in the number or conductance of postsynaptic receptors; this is distinct from previous work demonstrating changes in postsynaptic receptors mediating AMPAergic and GABAergic scaling in cultured neurons [10], [26], [27]. Our results suggested that embryonic motoneurons express synaptic scaling following activity blockade by simply increasing the driving force for all GABAergic synapses through chloride accumulation.

In the present study, we developed a faster less invasive technique of assessing Cl^−^_in_ for spinal neurons transfected with the genetic ratiometric chloride-indicator Clomeleon [11]. Using this technique to approximate Cl^−^_in_, we obtained the same values as those reported previously for control (motoneurons and interneurons) and lidocaine-treated (motoneurons) embryos using the perforated patch technique (∼50 mM - control, ∼85 mM – lidocaine [7], [18], [19]).

Studies from several different systems have indicated that high Cl^−^_in_ gradually decreases as the cells mature [28], [29]. Previous work in the embryonic mouse spinal cord showed a reduction of Cl^−^_in_ during development that was mediated by the reduced function of NKCC1 [30]. As described in the results, we see a reduction in Cl^−^_in_ from E8–10, however E8 values were still not as high as those in lidocaine-treated cells (p = 0.002); this suggests that the increased Cl^−^_in_ of activity-blocked motoneurons cannot simply be explained by a developmental delay in this process. Further, we have recently shown that the mechanism underlying AMPAergic scaling was not due to a developmental delay in the maturation of AMPA receptors [31]. While we only show a reduction of Cl^−^_in_ from E8 to E10, it is highly likely that Cl^−^_in_ continues to decline for several days. We know GABAergic potentials become less depolarizing at E14 and cannot be detected by E16–18 [32], consistent with the known maturation of Cl^−^ transporters which reduce Cl^−^_in_ during spinal development [33].

Increases in Cl^−^_in_ as a mechanism for scaling has been suggested in a previous study in hippocampal cultures where GABA was inhibitory [34]. Alternatively, whole cell recordings of cortical cultured pyramidal cells demonstrated homeostatic adjustments of mIPSC and mEPSC amplitude following activity blockade, and no change was observed in the reversal potentials [5], [35], [36].

The results demonstrate that chloride accumulation in embryonic motoneurons underlies the upward scaling of GABAergic mPSCs. However, we have not determined the underlying mechanism for the shift in E_Cl_. Several studies have shown that NKCC1, the Na^+^-K^+^-2Cl^−^ transporter, accumulates Cl^−^_in_ early in neural development [37]. In E10 chick motoneurons chloride appears to be accumulated by both NKCC1 and the anion exchanger (AE3) [19]. Therefore, NKCC1, AE3, or an unidentified chloride transporter could be functionally up-regulated. It remains unclear how the cell could increase Cl^−^_in_ by ≥30 mM. Charge considerations would not allow the cell to simply add 30 mM of an anion. Further, due to changes in osmolarity, it is unlikely that cells can add 30 mM of Cl^−^ and a cation to balance the charge. It may be possible that changes occur in other local anions [38].

### GABA_A_R blockade and spiking activity blockade both trigger scaling through chloride accumulation

Similar to blocking activity, blocking GABA_A_Rs *in ovo* from E8–E10 produced an upward scaling of GABAergic and AMPAergic quantal amplitude [8]. In fact, chronic GABA_A_R blockade increased mPSC amplitude about twice as much as activity blockade [6]. We therefore hypothesized that activity blockade produced scaling by reducing GABA release, thereby reducing GABA_A_R activation, which triggered synaptic scaling [8]. In this way, gabazine would be more effective in increasing mPSC amplitude than lidocaine because it directly blocked GABA_A_R activation associated with both evoked and spontaneous GABA release. If our hypothesis was correct we would expect that GABA_A_R blockade should produce scaling via the same mechanism as lidocaine, but do so more effectively. This does appear to be the case for AMPAergic scaling where increases in conductance mediated by the insertion of specific AMPAergic subunits were greater following GABA_A_R blockade than following activity-block [31]. In the current study, we show that the driving force for GABAergic currents is increased via chloride accumulation to the same extent following blockade of either activity or GABA_A_Rs (Fig. 4A & D). While this suggested the two treatments produced GABAergic scaling through the same mechanism (chloride accumulation), it did not explain why GABA_A_R block increased mPSC amplitude to a greater extent than activity blockade [8]. Therefore, unlike activity blockade, GABA_A_R block must trigger an additional increase in GABAergic mPSC conductance. This compensatory change in conductance may be triggered by the blockade of GABAergic spontaneous mPSCs, which would not be blocked in lidocaine-treated motoneurons.

These results highlight the importance of GABA_A_R activation in triggering homeostatic synaptic plasticity in embryonic motoneurons. The hypothesis that spiking activity triggers scaling through the associated reduction in GABA_A_R activation is thus supported by our observation that the mechanisms of scaling are similar following either activity or GABA_A_R block. It is possible that spiking activity is homeostatically maintained using GABA_A_R activation as a proxy for spiking activity, however 2 findings suggest that this may not be the case. First, upscaling does not occur in motoneurons by 12 hours of gabazine treatment, when the activity has recovered [8]. Second, 48 hour gabazine treatment triggered larger increases in mPSC amplitude but had no effect on SNA frequency, whereas lidocaine treatment generated smaller increases in mPSC amplitude but increased SNA frequency; the finding suggests there is not a simple relationship between quantal amplitude and SNA frequency. Alternatively, it is possible that compensatory upscaling may be a plasticity that acts to maintain synaptic strength.

Understanding which GABA_A_Rs trigger compensatory changes in synaptic strength will be important because these alterations in receptor activation occur in injury and disease. It is of interest that spinal cord injury, peripheral nerve injury, and traumatic brain injury produce depolarizing shifts in the chloride reversal potential, which will necessarily result in GABAergic scaling; this excitatory shift in E_Cl_ can result in a hyperexcitable pathophysiology [37], [39], [40], [41].

### Scaling through chloride accumulation is a common feature observed across many embryonic spinal neuron classes

Most studies on synaptic scaling focus on excitatory inputs to excitatory principle neurons. Far fewer studies have examined scaling in interneurons, and to date, the results have been varied. Some studies have found that excitatory inputs to inhibitory interneurons undergo scaling in a compensatory direction [42], [43], [44], however other studies do not find compensatory changes in the mEPSCs of inhibitory interneurons [5], [36], [45]. In one study where activity was reduced in 3 different classes of interneurons, no compensatory change was seen in either spontaneous EPSCs or IPSCs [46]. Another study using rat organotypic spinal cord cultures perturbed activity levels for several days and then assessed synaptic strength in ventral spinal interneurons. While these perturbations did not appear to produce scaling of mPSCs, spontaneous currents did appear to change in a compensatory direction [47]. In the present work, we were able to assess synaptic scaling through chloride accumulation across different interneurons. We found that following activity blockade or GABAergic blockade, scaling up via chloride accumulation was a common mechanism observed across what is likely to be various classes of interneurons and motoneurons. The distribution of chloride levels in control and treated spinal neurons were highly correlated through a multiplicative factor, suggesting the entire distribution of Cl^−^_in_ in different interneurons scaled upward (Fig 8).

The time course of the scaling process in motoneurons and interneurons seems to be different. Our data indicate that motoneuron chloride levels progressively increase from 24 to 48 hours of gabazine treatment, consistent with previous data [8]. However, interneuron chloride levels are increased after only 12 hours of GABA_A_R blockade. This raises the possibility that compensatory changes in GABAergic synaptic drive could contribute to the recovery of embryonic movements following *in ovo* GABA_A_R block, which occur within 12 hours [8].

The finding that virtually all components of the ventral spinal circuitry undergo a compensatory scaling up through chloride accumulation appears distinct from previous work showing variability among interneurons. We propose the generality of the scaling response may occur because of the early developmental stage of the network, where GABAergic, glutamatergic, and cholinergic components are all excitatory and recruit their postsynaptic partners. Enhancing the excitability of this more simplistic network could be achieved by increasing quantal strength across all the excitatory components. As GABA becomes inhibitory later in development synaptic scaling may become more limited as shown previously in the hippocampus [48]. Similarly spontaneous network activity in the developing retina homeostatically recovers within hours following GABAR blockade when GABA is depolarizing, but not later in development when GABA becomes hyperpolarizing [49]. It will be important to establish if these developmental plasticities are recovered following injury, and if other developing circuits also experience a more universal scaling process.
